# Unique Perspectives

**DOI:** 10.3201/eid2011.AC2011

**Published:** 2014-11

**Authors:** Jared Freidberg

**Affiliations:** Graduate Student, Georgia State University, Atlanta, Georgia, USA

**Keywords:** art science connection, emerging infectious diseases, art and medicine, about the cover, Paul Cezanne, Still Life with Apples, apples, unique perspectives, foodborne disease, foodborne outbreaks, food safety, post-impressionist, still life

**Figure:**
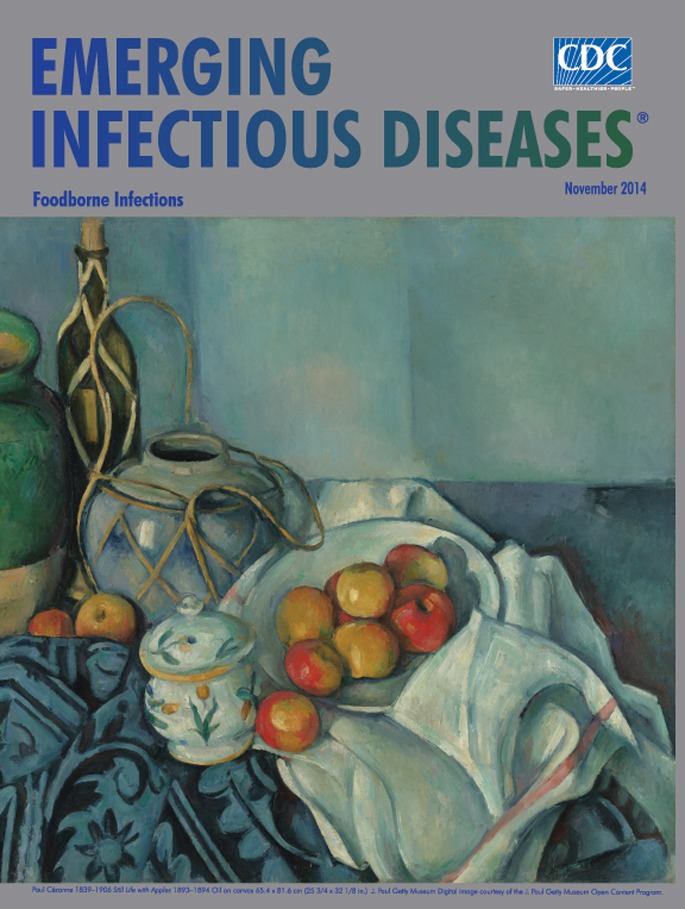
**Paul Cezanne (b. 1839) Still Life with Apples (1893–1894). Oil on canvas (25 3/4 × 32 1/8 in/65.4 × 81.6 cm).** Digital image courtesy of the Getty's Open Content Program, The J. Paul Getty Museum, Los Angeles, CA.

Paul Cezanne was a visionary who bridged 19th century impressionism with 20th century cubism and other modern styles. Born in Aix-en-Provence, France, into the family of a successful banker, Cezanne had a comfortable childhood, culminating in studies at the College Bourbon. There he met lifelong friends Emile Zola and Baptistin Baille, who would become prominent scholars in literature and acoustics, respectively.

Cezanne enrolled in the Free Municipal School of Drawing in Aix-en-Provence, but his father pressured him to enter law school to focus on more “practical” pursuits. Cezanne continued taking drawing lessons. Defying his father’s wishes, he moved to Paris in 1861 to join Zola and pursue becoming an artist. His father eventually softened to Cezanne’s career choice and granted him a large inheritance that freed Cezanne from financial uncertainty. Cezanne focused his early art on the whimsical landscapes of his childhood. As his style matured, he became captivated with defining paintings by simple shapes, exceptional lighting, and uncommon perspectives. By the 1890s, he had achieved artistic and financial success in Paris but chose to return to his native Provence where he preferred to paint.

*Still Life with Apples* (1893–1894) is a deconstruction of the impressionist art of the era. In this still life, the displayed apples monopolize the imagery, but their position on the table draws the gaze to the right. Cezanne overlaps and blends the vases and bottle, in a style that is in contrast to the full and complete forms championed by impressionists. The haphazard table cloths invoke the chaotic turbulence of ocean water, adding motion to the stillness of the painting. The red, green, and yellow hues of the apples emphasize their prominence against the softer colors of the table and vases. The perspective of the painting is also unique to Cezanne’s style: each piece of fruit, the bottle, the pots, and even the plate, retains an individual presence, giving them an independence from one another.

Cezanne’s interest in still life paintings was about the form, shade, and color of the presented objects. Cezanne stresses the shape and position of the apples over their functionality and appeal as a source for nourishment. The emphasis on shape allowed Cezanne to subvert the tropes of classical impressionism by demonstrating a unique viewpoint on how the apple’s form occupies its given space.

Cezanne asks people to look at the apples of his painting from a different perspective, and this may serve as a lesson for those who work to promote food safety. The food we eat may be touched by many people and rests on many surfaces before it comes to our plates. Our food may contact a multitude of microbes during its journey from farm or field to table. The capacity to detect, investigate, and prevent food-borne diseases varies across the world. Adapting surveillance processes and prevention techniques may require new approaches and unique perspectives, in much the way Cezanne implemented innovations to impressionism when it grew too limiting.
